# Synchronous Multiple Primary Cancers Involving Rectal Cancer and Diffuse Large B-cell Lymphoma of the Right Colon: A Case Report

**DOI:** 10.7759/cureus.83921

**Published:** 2025-05-11

**Authors:** Wei Ding, Hui Luo, Yufei Wang

**Affiliations:** 1 Department of Anorectal, The Affiliated Traditional Chinese Medicine Hospital of Southwest Medical University, Luzhou, CHN; 2 Department of Operations Management, Luzhou Traditional Chinese Medicine Hospital, Luzhou, CHN

**Keywords:** anterior resection, diffuse large b-cell lymphoma, rectal cancer, right-sided hemicolectomy, synchronous cancers

## Abstract

Colorectal cancer is a common malignant tumor of the digestive tract, but cases concurrently complicated with lymphoma are rare. This article reports a unique case of rectal adenocarcinoma combined with diffuse large B-cell lymphoma (DLBCL) in the right colon. An 82-year-old male was admitted to the hospital due to irregular bowel movements accompanied by abdominal pain. Abdominal CT and colonoscopy suggested a malignant tumor in the rectum. During laparoscopic anterior resection of the rectum, a firm mass measuring approximately 6 cm × 5 cm was observed in the mesentery posterior to the descending and horizontal segments of the duodenum, with unclear boundaries from the ileocecal region and ascending colon. Consequently, an open right hemicolectomy was simultaneously performed. Postoperative pathology revealed well-differentiated adenocarcinoma in the rectum, while the right colon tumor and intestinal segment, combined with immunohistochemistry, were consistent with diffuse large B-cell lymphoma (not otherwise specified (NOS)) of non-germinal center origin. This case provides important clinical insights: for patients with colorectal tumors, the possibility of multiple primary cancers should be considered, especially when symptoms and imaging findings are inconsistent. Multisite biopsies and molecular pathological analysis should be employed to reduce misdiagnosis.

## Introduction

Colorectal cancer and diffuse large B-cell lymphoma (DLBCL) are two distinct malignancies with entirely different pathogenic mechanisms and clinical treatment strategies. Although colorectal cancer ranks as the third most common malignancy globally, primary gastrointestinal DLBCL is relatively rare, accounting for only 1%-4% of gastrointestinal malignancies [1,2]. The coexistence of these two tumors in the same patient is exceedingly uncommon, with most reported cases involving dual lesions confined to the same intestinal segment. This rarity poses significant diagnostic challenges, overlapping symptoms such as abdominal pain, altered bowel habits, and weight loss, along with nonspecific imaging findings, often lead to misdiagnosis and delayed detection [3]. Furthermore, treatment strategies remain controversial due to potential conflicts between radical tumor resection and lymphoma-directed chemotherapy regimens [4]. This article presents a unique case of rectal adenocarcinoma coexisting with DLBCL in the right colon, aiming to enhance clinicians' awareness of this rare comorbidity and provide new insights for optimizing personalized treatment strategies.

## Case presentation

An 82-year-old male patient presented with a three-year history of increased bowel movements and aggravated symptoms accompanied by abdominal pain for one week. Approximately three years ago, the patient began experiencing frequent bowel movements (three to five times daily) with loose, yellowish stools of small volume, accompanied by mucus and rectal tenesmus. Over the past week, his symptoms worsened, with the onset of epigastric distending pain, acid reflux, and heartburn. Upon evaluation at a local hospital, contrast-enhanced computed tomography (CT) of the chest and abdomen revealed a malignant rectal tumor. The patient had undergone a "nephrolithotomy" more than 20 years ago. Digital rectal examination identified a cauliflower-like mass approximately 6 cm from the anal verge, occupying more than half of the rectal circumference with limited mobility.

**Figure 1:**
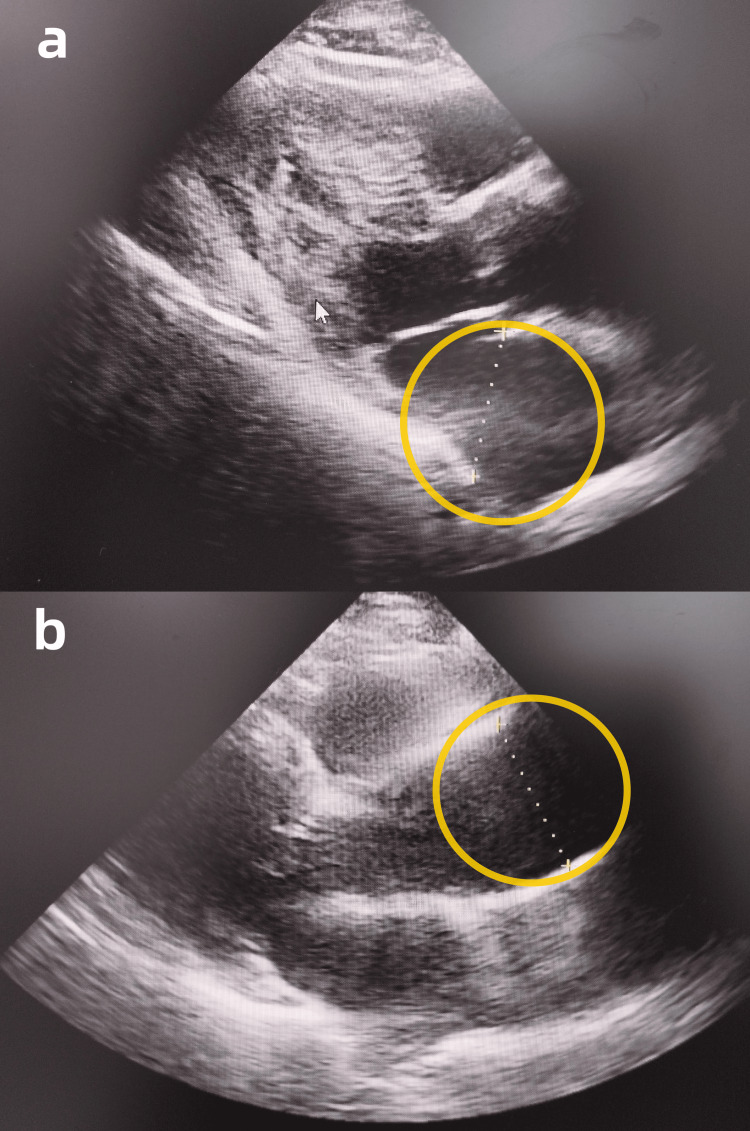
Echocardiography (a) The yellow circle indicates mild left atrial enlargement. (b) The yellow circle indicates a dilated ascending aorta.

**Figure 2:**
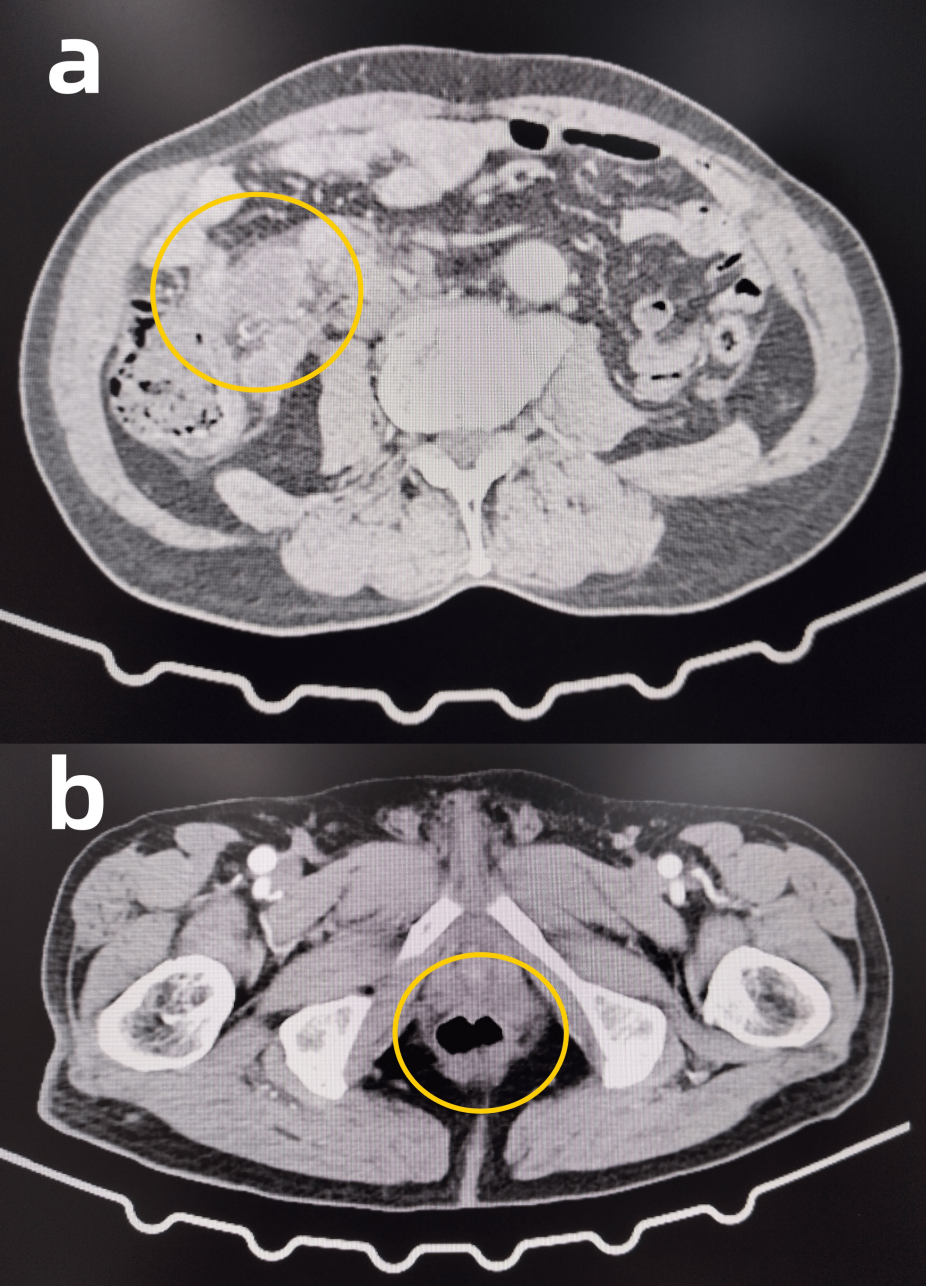
Axial CT scan of the abdomen (a)The yellow circle indicates mass in the right colon and mesenteric region. (b)The yellow circle indicates a rectum mass. CT: computed tomography

**Figure 3:**
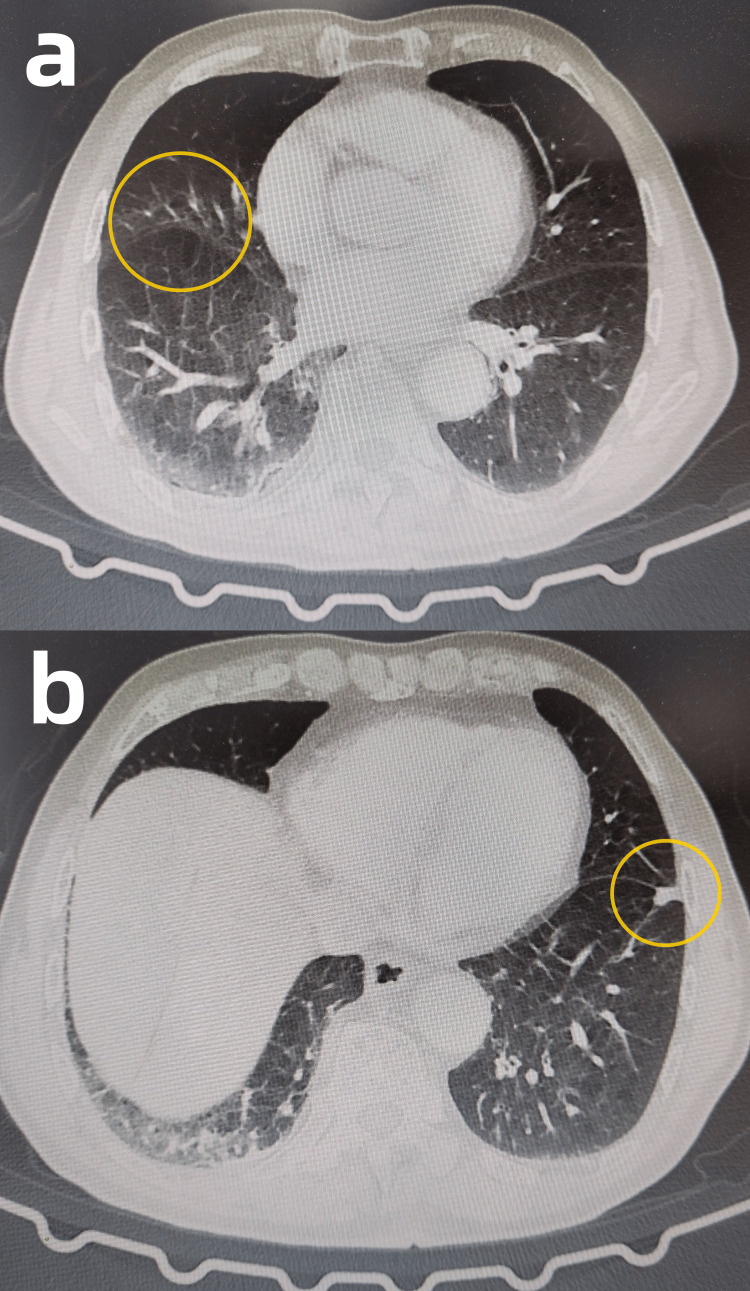
Axial CT scan of the chest (a) The yellow circle indicates pulmonary emphysema and mild inflammation in the right middle lobe. (b) The yellow circle indicates a solid nodule in the anteromedial basal segment of the left lower lobe. CT: computed tomography

**Figure 4:**
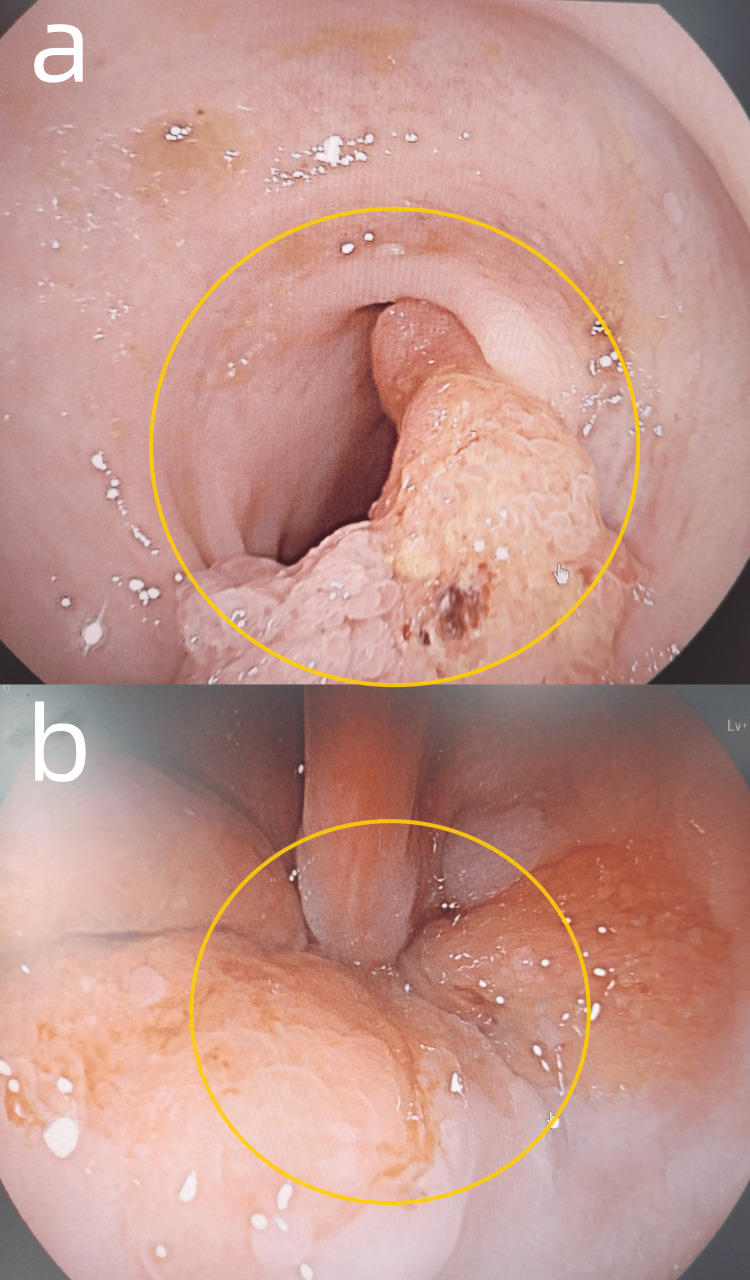
Colonoscopy and gastroscopy (a) The yellow circle indicates a rectal tumor. (b) The yellow circle indicates cardiac laceration, chronic gastritis.

Some relevant auxiliary examinations are presented as follows. Hemoglobin was measured at 124 g/L in this patient’s complete blood count (Reference range: 130-175g/L). Electrocardiogram (ECG) findings were within normal limits. Echocardiography revealed left atrial enlargement and dilated ascending aorta (Figure 1a, 1b). Abdominal contrast-enhanced CT imaging demonstrated a mass in the upper rectum, suggestive of a neoplastic lesion, and multiple enlarged lymph nodes in the left retroperitoneum and right mesenteric region (differential diagnosis: metastatic vs. inflammatory) (Figure 2a, 2b). Chest contrast-enhanced CT demonstrated pulmonary emphysema, mild inflammation in the right middle lobe, and a solid nodule in the anteromedial basal segment of the left lower lobe (Figure 3a, 3b). Diagnostic colonoscopy disclosed rectal tumor and sigmoid colon polyp, and gastroscopy disclosed cardia laceration, chronic non-atrophic gastritis with erosion, gastric polyp, and duodenitis. (Figure 4a, 4b).

After a comprehensive evaluation and exclusion of surgical contraindications, the patient was scheduled for laparoscopic exploration combined with laparoscopic anterior resection under general anesthesia. Following satisfactory anesthesia, the patient was placed in a modified lithotomy position with Trendelenburg tilt. A 10-mm optical trocar was inserted 0.5 cm above the umbilicus. One 15-mm main operating port was placed 2 cm medial to the right anterior superior iliac spine (ASIS), and three 5 mm auxiliary ports were established at the following sites: (1) 2 cm lateral to the right rectus abdominis at the umbilical level, (2) 1 cm below the umbilicus along the left rectus abdominis border, and (3) the lateral one-third point of the line connecting the left ASIS and umbilicus. Laparoscopic exploration revealed adhesions between the sigmoid colon and the left abdominal wall. The primary rectal tumor, located below the peritoneal reflection, was not visualized laparoscopically. A firm 6 cm × 5 cm mass was identified posterior to the descending and horizontal portions of the duodenum, involving the mesentery with ill-defined borders adjacent to the ileocecal region and ascending colon.

**Figure 5:**
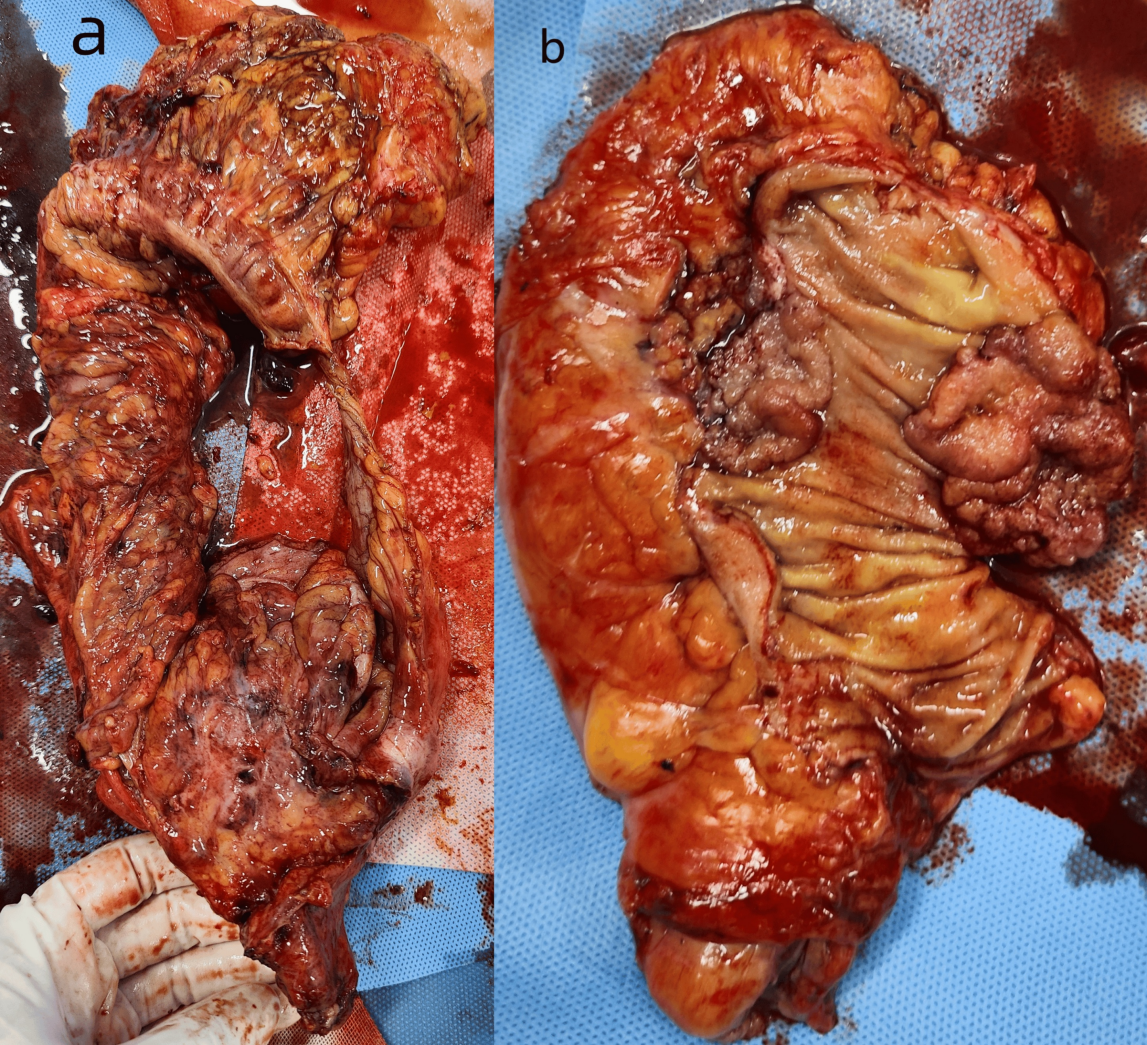
Resected specimen (a)Right hemicolectomy specimen. (b)Proctectomy specimen.

The surgical steps are as follows: 1. Adhesiolysis of the sigmoid colon was performed. The sigmoid mesentery was incised along the avascular plane (white line of Toldt’s), extending inferiorly to the peritoneal reflection and superiorly to the root of the small bowel mesentery. The inferior mesenteric artery (IMA) was isolated, clipped, and transected. The inferior mesenteric vein (IMV) was similarly ligated. 2. Total mesorectal excision (TME) was carried out 2 cm distal to the tumor. The bowel was skeletonized 10 cm proximal and 2 cm distal to the tumor. A linear stapler was introduced through the 15 mm right lower quadrant port to transect the rectum 2 cm below the tumor. 3. The left lower quadrant port site was extended to 5 cm for specimen retrieval (protected with a wound retractor). The resected segment (10 cm proximal to the tumor) and a sigmoid polyp were sent for pathology. A purse-string suture was placed in the proximal colon to secure the anvil of the circular stapler. After re-establishing pneumoperitoneum, a transanal stapled colorectal anastomosis was completed. 4. Due to the large, poorly demarcated mesenteric mass near the right colon, laparoscopic resection was deemed unsafe. A midline laparotomy was performed. The retroperitoneum was incised along the superior mesenteric vessels, with en bloc lymphadenectomy of the root of the superior mesenteric artery. The ileocolic, right colic, and right branches of the middle colic vessels were skeletonized and ligated. The hepatic flexure and right lateral peritoneum were fully mobilized. 5. The ileocecal region, ascending colon, proximal transverse colon, and mesenteric mass were resected en bloc. A side-to-side ileotransverse anastomosis was created using a linear stapler (reinforced with 3-0 absorbable sutures). A prophylactic loop ileostomy was fashioned 25 cm proximal to the anastomosis. 6. Two drains were placed in the right paracolic gutter and pelvic cavity. The abdomen was closed in layers, and the ileostomy was matured. The resected specimen was sent for pathological examination (Figure 5a, 5b).

**Figure 6:**
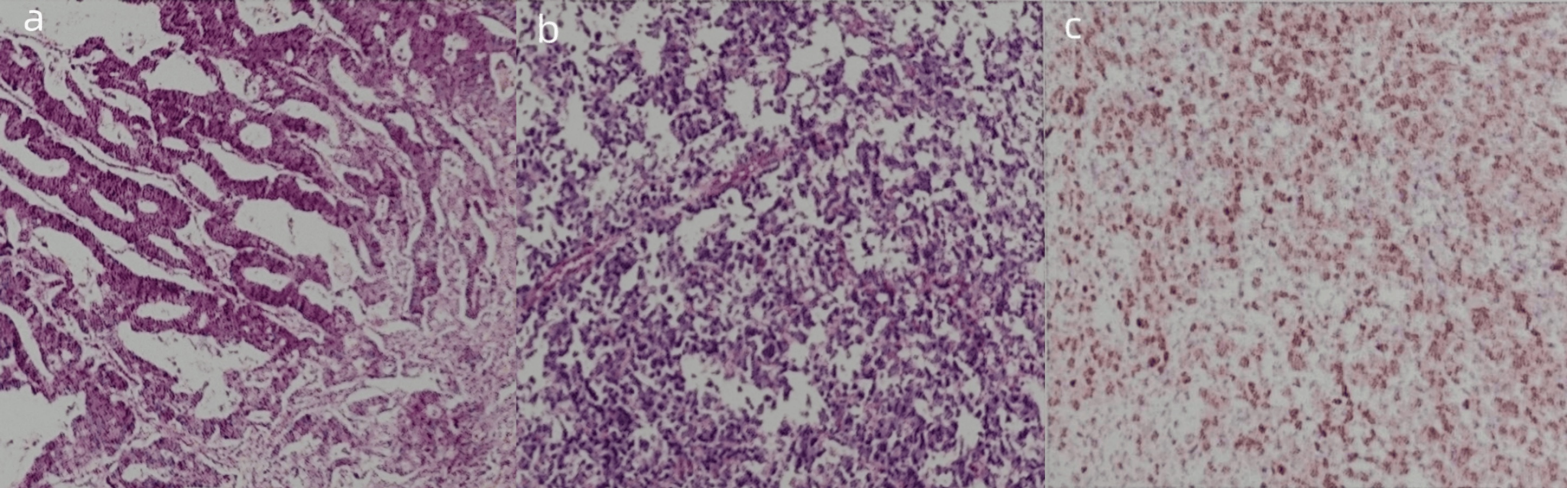
Representative H&E stain of the resected specimen (a) Rectal adenocarcinoma. (b) Right colon diffuse large B-cell lymphoma. (c) Lymphoma cells. H&E: hematoxylin and eosin

Postoperative pathological examination report (Figure 6): 1. Rectal tumor segment: Histological and gross type: Adenocarcinoma (not otherwise specified (NOS)), exophytic type. Histological grade: Well-differentiated. Tumor size: 4 cm× 5 cm × 1.5 cm. Depth of invasion: Involving perirectal tissues. Tumor budding grade: High. Lymphovascular invasion: Absent. Perineural invasion: Absent. Margin status: Both "proximal and distal margins" and "circumferential resection margin" were free of carcinoma. Lymph nodes: No enlarged lymph nodes detected. 2. Right colon tumor and intestinal segment: Combined with immunohistochemistry, findings were consistent with diffuse large B-cell lymphoma (DLBCL, NOS), non-germinal center origin. Tumor size: Approximately 3 cm × 2 cm × 0.8 cm. Tumor involvement: Full-thickness invasion of the intestinal wall. Margin status: Both resection margins were free of tumor. Mesenteric lymph nodes: 22 lymph nodes were examined, all showing lymphoma involvement. Appendix: No tumor invasion detected. 3. Submitted "sigmoid colon polyp": Mucosal tubular adenoma. 4. Submitted "root lymph node of the superior mesenteric artery": Six enlarged lymph nodes were identified, all containing lymphoma cells. Immunohistochemistry (Case 4#): SATB-2 (+), Ki67 (+, 40%), D2-40 (lymphatic vessels, +), S-100 (nerves, +), MLH1 (+), PMS2 (+), MSH2 (+), MSH6 (+). Immunohistochemistry (Case 14#): CD3 (-), CD5 (-), CD20 (diffuse, +), CD79a (diffuse, +), CD21 (FDC network), CD30 (-), Ki-67 (+, 80%), PAX-5 (+), BCL-2 (+), BCL-6 (+), CD10 (-), MUM-1 (+), CD138 (-), CD38 (+), C-MYC (+, 10%).

The patient was discharged after satisfactory postoperative recovery but did not undergo further treatment in the oncology department. A two-month postoperative telephone follow-up revealed that the patient reported poor appetite, occasional abdominal pain, but normal stoma function with adequate passage of gas and stool.

## Discussion

Colorectal cancer represents one of the most prevalent malignant tumors in the gastrointestinal tract, with adenocarcinoma being its predominant histological subtype. However, cases of synchronous primary colorectal adenocarcinoma and lymphoma are exceptionally rare, particularly those coexisting with primary DLBCL. Primary colorectal lymphoma itself is an uncommon malignancy, constituting merely 0.2% of all neoplasms in the colorectal region, with preferential involvement of the cecum, ascending colon, and rectum [5]. This disease entity predominantly affects male patients between 40 to 60 years of age, typically manifesting with abdominal pain, weight loss, palpable abdominal masses, or lower gastrointestinal bleeding.

This case report presents a rare instance of coexisting rectal adenocarcinoma and right colon DLBCL, highlighting the clinicopathological characteristics that warrant in-depth investigation into the mechanisms underlying dual malignancies and the associated diagnostic and therapeutic challenges. Although both colorectal adenocarcinoma and lymphoma are common gastrointestinal tumors, their synchronous occurrence in the same patient is exceedingly rare. Previous literature has primarily documented cases of colorectal adenocarcinoma combined with mucosa-associated lymphoid tissue (MALT) lymphoma, while reports of concurrent aggressive DLBCL are even more uncommon [6]. This phenomenon suggests that chronic inflammation or immune microenvironment dysregulation may play a potential role in the co-occurrence of these two malignancies. For instance, prolonged intestinal inflammation might simultaneously promote adenocarcinoma proliferation and induce clonal B-cell expansion through activation of the NF-κB pathway [7].

Diagnostically, the coexistence of these two malignancies increases the risk of a missed diagnosis. DLBCL may present radiologically as bowel wall thickening or mass lesions, which can overlap morphologically with adenocarcinoma features [8]. In the present case, preoperative colonoscopy only performed biopsies on the rectal lesion, while the right colon lesion was overlooked due to the absence of typical ulceration or protrusion until postoperative pathology unexpectedly revealed the DLBCL component. This underscores the need for heightened clinical vigilance toward multiple primary cancers, particularly in patients with extensive lesions or atypical presentations. Expanding the scope of endoscopic biopsies and incorporating immunohistochemical analysis (e.g., CD20, CD3, and CK markers) is essential for accurate diagnosis. Notably, the DLBCL in this case demonstrated strongly CD20-positive tumor cells with a remarkably high Ki-67 proliferation index of 80%, indicative of highly aggressive biological behavior.

The coexistence of dual primary malignancies presents significant challenges in therapeutic strategy formulation. While surgical resection remains the standard treatment for colorectal adenocarcinoma, DLBCL primarily requires chemotherapy (e.g., R-CHOP regimen) or targeted therapy [9]. When preoperative CT reveals a suspicious lymphomatous mass in a patient with coexisting lymphoma and adenocarcinoma, treatment strategy optimization requires a multidisciplinary approach integrating precise diagnosis, staging accuracy, and tailored therapeutic sequencing. First, distinguishing between metastatic lymphadenopathy from rectal cancer and primary lymphoma is critical, as their biological behaviors and treatment paradigms differ substantially. CT imaging, while effective in delineating tumor size, local invasion, and distant metastasis (e.g., hepatic involvement), has limited specificity for differentiating lymphoma from metastatic adenopathy. Complementary modalities such as PET-CT or MRI may enhance diagnostic accuracy by evaluating metabolic activity and soft-tissue resolution, respectively. Histopathological confirmation via endoscopic or percutaneous biopsy remains mandatory to confirm lymphoma diagnosis, as misdiagnosis risks inappropriate surgical intervention. For coexisting malignancies, treatment sequencing should prioritize addressing the biologically aggressive component. For instance, if lymphoma is confirmed, neoadjuvant chemotherapy (e.g., R-CHOP regimen) may be initiated to reduce tumor burden, followed by surgical resection of the rectal primary and residual colonic lesions. Conversely, if rectal adenocarcinoma dominates, upfront resection with en bloc lymphadenectomy (e.g., right hemicolectomy with D3 lymph node dissection) may be warranted, particularly if lymphoma is localized and responsive to subsequent systemic therapy. Surgical planning must account for the anatomical complexity of dual pathologies. Extended right hemicolectomy with meticulous mesocolic excision ensures adequate margins for both malignancies, while avoiding iatrogenic injury to adjacent structures (e.g., duodenum or ureters). Postoperative adjuvant therapy should integrate regimens targeting both entities, such as fluoropyrimidine-based chemotherapy for adenocarcinoma and immunochemotherapy for lymphoma, guided by molecular profiling and residual disease status. In conclusion, a tailored, stepwise strategy emphasizing accurate histopathological diagnosis, multidisciplinary collaboration, and individualized therapeutic sequencing is essential to optimize oncological outcomes in this rare clinical scenario. In the present case, DLBCL diagnosis was established postoperatively through specimen examination, as it was not identified preoperatively. Typically, for patients presenting with colorectal tumor obstruction, a multidisciplinary team (MDT) approach may prioritize radical resection to relieve obstruction, followed by systemic therapy for DLBCL, a strategy that balances tumor burden control with treatment toxicity minimization [10]. However, the impact of chemotherapy on postoperative intestinal function recovery and the potential for radiotherapy to exacerbate intestinal damage warrant long-term follow-up evaluation. Notably, emerging evidence suggests certain DLBCL patients may benefit from PD-1 inhibitors, but the applicability of immunotherapy in cases with concurrent solid tumors and its potential synergistic effects with adenocarcinoma-targeted therapies require further investigation [11].

This case has certain limitations, including the lack of whole-exome sequencing to identify potential shared driver gene mutations in both tumors, as well as a relatively short follow-up duration with incomplete long-term survival data. Nevertheless, it provides valuable clinical insights: for patients with colorectal tumors, clinicians should maintain a high index of suspicion for multiple primary malignancies, particularly when clinical symptoms and imaging findings are discordant. Multisite biopsies combined with molecular pathological analysis should be performed to minimize diagnostic oversight. Furthermore, the development of personalized treatment strategies must comprehensively consider tumor biological behavior, patient tolerance, and potential therapeutic interactions.

## Conclusions

This case report of synchronous primary malignancies, rectal adenocarcinoma combined with right colon DLBCL, highlights a rare and clinically challenging co-occurrence of distinct pathological entities. Although the precise pathogenesis remains incompletely understood, potential contributing factors may include genetic predisposition, immune microenvironment dysregulation, and shared carcinogenic exposures. The diagnostic challenge in this case stemmed from overlapping clinical presentations and partially concordant imaging findings, necessitating a multidisciplinary approach (incorporating endoscopic, radiologic, and pathologic correlation) with immunohistochemical characterization (CD20, CD3, Ki-67, etc.) for accurate differentiation. Therapeutically, optimal management requires careful consideration of both surgical resection for the rectal carcinoma and R-CHOP chemotherapy for DLBCL, with particular attention to treatment sequencing, potential synergies, and patient tolerance. This case underscores the importance of maintaining clinical vigilance for multiple primary tumors when evaluating colorectal masses to prevent diagnostic oversight. Future research should focus on elucidating the molecular mechanisms underlying such dual malignancies and refining personalized treatment strategies to improve patient outcomes and quality of life.
